# AI-driven predictive modeling of supercritical CO_2_ solubility using cheetah-optimized machine learning

**DOI:** 10.3389/fmed.2026.1973985

**Published:** 2026-10-14

**Authors:** Almuhannad S. Alorfi, Nidal H. Abu-Hamdeh

**Affiliations:** 1 Department of Information Systems, Faculty of Computing and Information Technology, King Abdulaziz University, Jeddah, Saudi Arabia; 2 Center of Research Excellence in Renewable Energy and Power Systems/Energy Efficiency Group, Department of Mechanical Engineering, Faculty of Engineering, King Abdulaziz University, Jeddah, Saudi Arabia

**Keywords:** artificial intelligence, cheetah optimizer, data-driven modeling, Gaussian process regression, hyperparameter optimization, machine learning, predictive modeling, supercritical CO^2^

## Abstract

Artificial intelligence (AI) and machine-learning models provide powerful data-driven tools for modeling complex nonlinear systems and predicting process behavior from limited experimental data. In this study, an AI-driven predictive framework is developed for modeling paracetamol solubility and solvent density in supercritical carbon dioxide (SC-CO_2_). A dataset comprising 40 experimental observations was employed, with pressure and temperature used as input variables and paracetamol mole fraction and SC-CO_2_ density considered as target outputs. Three machine-learning regression algorithms—Gaussian Process Regression (GPR), Tweedie Regression (TDR), and K-Nearest Neighbors (KNN)—were developed and comparatively evaluated. To enhance predictive performance and automate model configuration, the Cheetah Optimizer (CO), a nature-inspired metaheuristic optimization algorithm, was integrated into the modeling framework for hyperparameter optimization. Model performance was assessed using the coefficient of determination (*R*^2^), mean squared error (MSE), and mean absolute error (MAE). The results demonstrated that TDR provided the highest predictive accuracy for paracetamol mole fraction, achieving an *R*^2^ of 0.985, whereas GPR exhibited the best performance for SC-CO_2_ density prediction, with an *R*^2^ of 0.991. The developed framework successfully captured the nonlinear relationships between operating conditions and the predicted properties while demonstrating that the optimal learning algorithm depends on the target variable. These findings highlight the potential of AI-assisted optimization and machine-learning approaches for data-driven prediction and intelligent modeling of complex supercritical-fluid processes.

## 1 Introduction

Production of nanomedicines can be carried out via different methods to enhance the solubility of pharmaceuticals in aqueous media. Since solubility enhancement has been one of challenges in the area of pharmaceutical industry, drug solubility enhancement is a great topic of interest. Mechanical and chemical methods can be used for nanonization of drug particles of solid dosage oral formulations to reduce their side effects by reducing the dosage (1, 2). When the drug is nanosized, its solubility in aqueous environment would be drastically enhanced meaning that less drug dosage should be taken by patients which in turn results in less adverse effects of drugs. So, nanosized drugs would be a great idea to improve the drugs' efficacy and patient compliance.

The methods based on supercritical technique are reported recently as an alternative to other nano-based processing for drug particles nanonization (3, 4). The method is straightforward and can be classified as a top-down approach for nanonization of drugs where drug particles at micron size are converted to sub-micron sized drug particles by the aid of supercritical solvents such as supercritical CO_2_ (SC-CO_2_). Rapid expansion of supercritical solution (RESS) is one of the popular methods which can be conducted using supercritical methods (5–7). The method is facile and can be done in continuous mode by the aid of supercritical CO_2_ as the solvent of process. So, the solution contains supercritical solvent and the dissolved drug. If the drug is not soluble enough in the solvent, it would not be suitable for RESS processing to prepare nanosized drugs. Therefore, solubility measurements and also correlations would be essential and as the first step for development of RESS for solid-dosage formulations (8, 9).

Drug solubility in supercritical carbon dioxide (SC-CO_2_) can be evaluated through direct experimental measurements or estimated using theoretical and computational correlations. Experimental data are essential for characterizing phase behavior and for developing and validating predictive frameworks that can reduce the time, cost, and resources required for extensive solubility measurements. Thermodynamic and machine-learning approaches are among the most widely applied methods for correlating and predicting drug solubility in SC-CO_2_ (10–13). In particular, recent advances in machine learning have increased their application in this field because of their ability to capture complex, nonlinear relationships between solubility and operating variables. Accordingly, the present study develops an AI-driven predictive framework that integrates machine-learning models with metaheuristic optimization to accurately predict drug solubility in SC-CO_2_ under different pressure and temperature conditions.

AI-based predictive modeling provides an effective computational approach for capturing complex relationships between operating conditions and process outputs. In supercritical-fluid processing, accurate prediction of drug mole fraction and solvent density is particularly important because these properties directly influence process performance and product characteristics. Nevertheless, reliable estimation remains challenging owing to the structural complexity of pharmaceutical compounds and the nonlinear interactions occurring between solutes and solvents under different operating conditions. Machine-learning methods offer a promising alternative to conventional empirical and theoretical approaches because they can identify complex relationships directly from available experimental data. As a branch of artificial intelligence, machine learning employs computational algorithms and statistical models to learn patterns from previously collected data and subsequently generate predictions or support decision-making (14, 15). Consequently, ML techniques have been increasingly applied to the prediction and modeling of complex properties and phenomena across medicine, chemistry, pharmaceutical sciences, and related disciplines.

A specific research gap remains at the intersection of paracetamol/SC-CO_2_ modeling and AI-assisted model configuration. Previous studies have established the importance of experimental and computational solubility estimation, but a target-specific framework that jointly evaluates paracetamol mole-fraction solubility and SC-CO_2_ density using GPR, TDR, and KNN while applying Cheetah Optimizer-based hyperparameter tuning has not been clearly established for this dataset. This comparison is useful because the two target variables have different numerical scales and response characteristics, so the model that is most effective for solubility need not be the one that is most effective for density (16).

The objective of the present investigation was to develop an AI-assisted computational framework for predicting paracetamol mole-fraction solubility and SC-CO_2_ density by integrating machine-learning regression models with metaheuristic hyperparameter optimization. The models were trained and tested on a dataset of 40 samples with the input features Pressure and Temperature. We used metrics such as R-squared, MSE, and MAEs to compare the selected models: Gaussian Process Regression (GPR), Tweedie Regression (TDR), and K-Nearest Neighbors (KNN). For hyper-parameter optimization, the Cheetah Optimizer (CO) was used to find the best values for the model parameters. These models are used and tuned for the first time to correlate the solubility of paracetamol in supercritical carbon dioxide.

Gaussian Process Regression (GPR) is a probabilistic model that can capture complex patterns in the data and create reliable predictions. K-Nearest Neighbors (KNN) is also a non-parametric model that relies on similarities between data points to make predictions. Tweedie Regression (TDR) is a generalized linear model that can handle data with heavy-tailed distributions. In this research, we evaluated the effectiveness of these three algorithms to determine which one is the most suitable for predicting mole fraction and density.

Accordingly, the principal contributions of this study are fourfold: (i) simultaneous prediction of paracetamol mole-fraction solubility and SC-CO_2_ density from pressure and temperature; (ii) comparative evaluation of GPR, TDR, and KNN regression models for two distinct target variables; (iii) integration of the Cheetah Optimizer for hyperparameter optimization within the predictive framework; and (iv) physical interpretation of the predicted pressure- and temperature-dependent trends to assess whether the model behavior is consistent with the expected supercritical-fluid response within the investigated operating domain.

## 2 Drug solubility data in the solvent

The dataset employed in this study was obtained from Ref (17). and comprises experimental measurements of paracetamol solubility, expressed as the drug mole fraction, together with the density of supercritical CO_2_ under different operating conditions. Temperature (T) and pressure (P) were used as the input variables, whereas the dimensionless mole fraction of paracetamol and solvent density were considered the target outputs. The dataset consists of 40 observations covering temperature and pressure ranges of 311–358 K and 95–265 bar, respectively, with the density and mole-fraction values reported in scientific notation. All experimental observations are presented in Table 1, while Figure 1 illustrates the relationships among the input and output variables using scatter plots. These graphical representations provide an initial assessment of the variations and underlying trends in the dataset, which can subsequently be compared with the model predictions to evaluate the reliability of the developed solubility-prediction framework. Alotaibi et al. (16) used the same data of solubility in building some ML models, and authors applied their ML methodology for this study.

**Table 1:** Experimental data on drug solubility (16, 17).

T (K)	P (bar)	Mole fraction	Density
311	95	4.30 × 10^−7^	630.0973
311	110	5.50 × 10^−7^	709.3039
311	120	6.40 × 10^−7^	738.934
311	140	8.80 × 10^−7^	779.9198
311	170	1.04 × 10^−6^	821.3081
311	190	1.20 × 10^−6^	842.2725
311	205	1.37 × 10^−6^	855.8614
311	225	1.70 × 10^−6^	871.9051
311	240	1.84 × 10^−6^	882.7209
311	265	2.01 × 10^−6^	898.951
323	95	3.90 × 10^−7^	335.9467
323	110	4.60 × 10^−7^	504.583
323	120	1.01 × 10^−6^	583.4263
323	140	1.10 × 10^−6^	671.7236
323	170	1.20 × 10^−6^	741.3601
323	190	2.15 × 10^−6^	772.1935
323	205	2.36 × 10^−6^	791.1074
323	225	2.63 × 10^−6^	812.5842
323	240	2.96 × 10^−6^	826.6273
323	265	3.16 × 10^−6^	847.1526
348	95	3.50 × 10^−7^	215.265
348	110	4.10 × 10^−7^	274.5146
348	120	1.34 × 10^−6^	320.3364
348	140	2.23 × 10^−6^	419.5845
348	170	4.24 × 10^−6^	544.6907
348	190	6.21 × 10^−6^	603.3571
348	205	6.92 × 10^−6^	637.8761
348	225	7.65 × 10^−6^	675.1868
348	240	9.38 × 10^−6^	698.4607
348	265	9.89 × 10^−6^	730.9669
358	95	3.10 × 10^−7^	196.2869
358	110	3.60 × 10^−7^	244.2486
358	120	3.05 × 10^−6^	280.0767
358	140	4.12 × 10^−6^	359.0505
358	170	6.17 × 10^−6^	475.0856
358	190	8.58 × 10^−6^	537.8208
358	205	1.02 × 10^−5^	576.5307
358	225	1.20 × 10^−5^	619.1964
358	240	1.51 × 10^−5^	645.9861
358	265	1.64 × 10^−5^	683.3597

**Figure 1:**
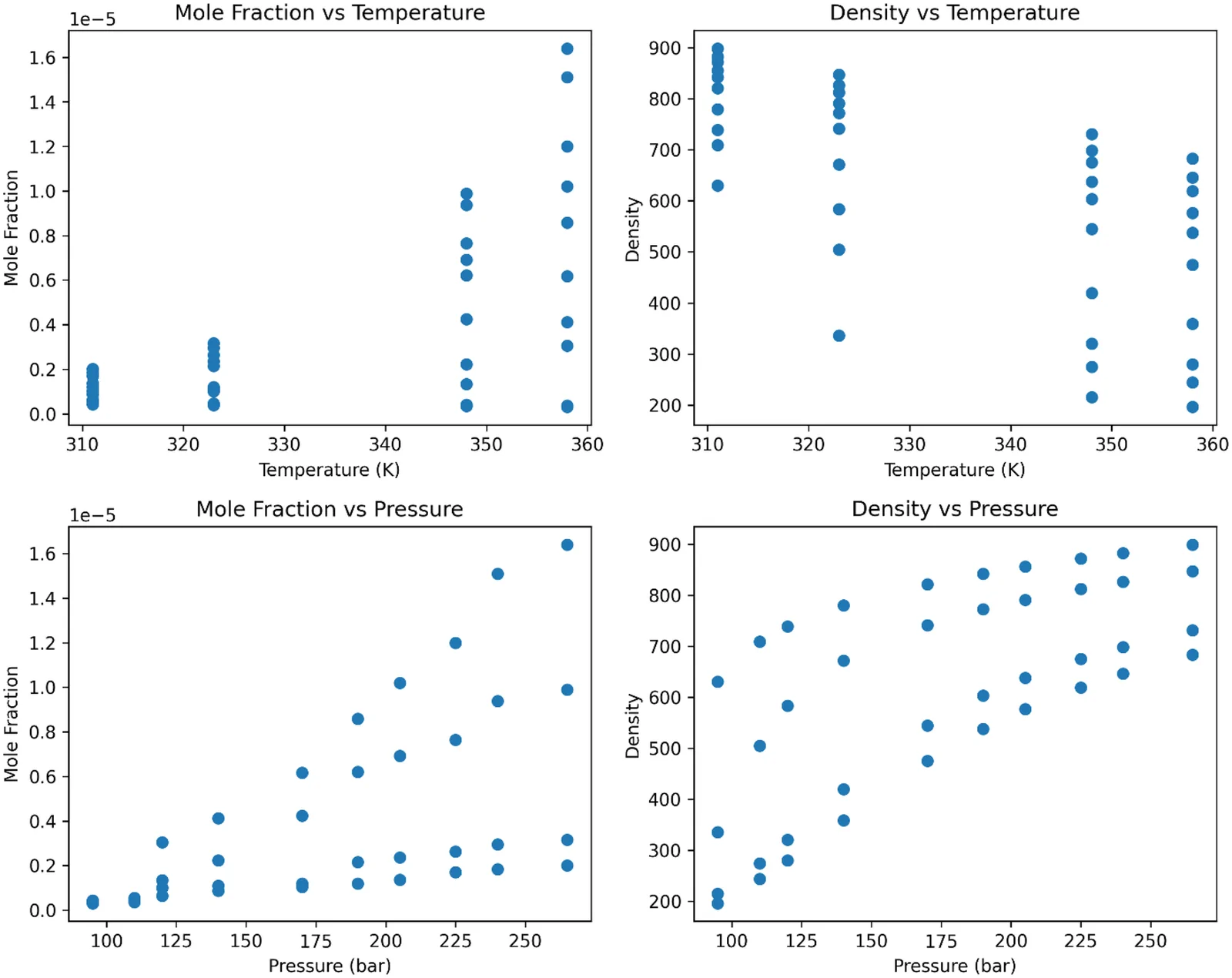
Scatter plots of input and output variables against each other.

## 3 Methodology

### 3.1 Cheetah optimizer (CO)

The Cheetah Optimizer (CO) is a population-based, derivative-free metaheuristic inspired by the principal hunting behaviors of cheetahs: searching, sitting and waiting, attacking, and abandoning an unsuccessful hunt before returning to the home range (18, 19). These behavioral modes are translated into stochastic position-update rules that enable the algorithm to alternate between global exploration and local exploitation. Unlike gradient-based optimization, CO does not require derivatives or an explicit analytical form of the objective function; therefore, it is suitable for hyperparameter-tuning problems in which the relationship between the candidate hyperparameters and predictive performance is nonlinear, discontinuous, or available only through repeated model training and validation (19).

For a problem with *D* decision variables, the position of the *i*th cheetah at iteration *t* represents a candidate hyperparameter vector (18):

$$\theta_{i}^{t}=[\theta_{i,1}^{t},\theta_{i,2}^{t},\ldots ,\theta_{i,D}^{t}],\;i=1,2,\ldots ,n,$$ (1)

where *n* is the population size and each component is restricted by its lower and upper bounds, $L_{j}\leq \theta_{i,j}\leq U_{j}$. In the present regression framework, the quality of each candidate can be quantified using the validation mean-squared error:

$$F(\theta_{i}^{t})=\frac{1}{N_{v}}\overset{N_{v}}{\underset{q=1}{\sum }}[y_{q}-{\hat{y}}_{q}(\theta_{i}^{t}){]}^{2},$$ (2)

where $N_{v}$ is the number of validation observations, $y_{q}$ is the experimental response, and ${\hat{y}}_{q}(\theta_{i}^{t})$ is the prediction produced by the model configured with $\theta_{i}^{t}$. Accordingly, hyperparameter optimization is expressed as $\theta^{*}=\mathrm{argmi}n_{\theta }F(\theta )$. When cross-validation is used, *F* can equivalently be defined as the mean validation loss over the selected folds, thereby reducing the dependence of the optimization result on a single data partition.

During the search mode, a cheetah changes its position stochastically to examine unexplored regions of the admissible solution space. For the *j*th decision variable, the update is written as (18):

$$\theta_{i,j}^{t+1}=\theta_{i,j}^{t}+{\hat{r}}_{i,j}^{\;-1}\alpha_{i,j}^{t},$$ (3)

where ${\hat{r}}_{i,j}$ is a normally distributed randomization parameter and $\alpha_{i,j}^{t}>0$ denotes the adaptive step length. The stochastic term diversifies the population, whereas the step length regulates the magnitude of movement and may be scaled according to the search bounds, iteration number, or distance from a neighboring agent. In the sitting-and-waiting mode, the candidate remains unchanged:

$$\theta_{i,j}^{t+1}=\theta_{i,j}^{t}.$$ (4)

This stationary mode prevents all agents from being modified simultaneously and preserves potentially useful solutions while other population members explore alternative regions.

The attack mode intensifies the search around the best solution identified so far. Its position-update rule is (18):

$$\theta_{i,j}^{t+1}=\theta_{B,j}^{t}+{ř}_{i,j}\beta_{i,j}^{t},$$ (5)

where $\theta_{B,j}^{t}$ is the *j*th component of the current global-best solution, $\beta_{i,j}^{t}=\theta_{k,j}^{t}-\theta_{i,j}^{t}$ is an interaction factor defined using a randomly selected neighboring cheetah k≠i, and ${ř}_{i,j}$ is the turning factor:

$${ř}_{i,j}=|r_{i,j}{|}^{\exp(r_{i,j}/2)}\sin(2\pi r_{i,j}),\;r_{i,j}∼N(0,1).$$ (6)

This formulation directs the population toward the most promising region while retaining randomized directional changes, which helps maintain diversity during exploitation. The selection of search, waiting, or attack behavior is controlled probabilistically. A time-dependent switching variable can be expressed as

$$H=\exp\left[2\left(1-\frac{t}{T}\right)\right](2r_{1}-1),$$ (7)

where *T* is the maximum hunting duration and $r_{1}∼U(0,1)$. Together with additional random thresholds, *H* regulates transitions among the behavioral modes and consequently the balance between exploration and exploitation. After each update, infeasible components are returned to the admissible domain using

$$\theta_{i,j}^{t+1}\leftarrow \min(U_{j},\max(L_{j},\theta_{i,j}^{t+1})).$$ (8)

Through the coordinated use of stochastic searching, solution preservation, best-solution-guided attack, and restart behavior, CO provides a systematic balance between broad exploration and focused exploitation for model hyperparameter optimization (18, 19).

### 3.2 Tweedie regression (TDR)

Tweedie regression is a type of generalized linear model (GLM) that is used to model data with a Tweedie distribution, which is a flexible family of distributions that includes gamma, poisson, and inverse Gaussian distributions (16). The variance-mean relationship of the Tweedie distribution is controlled by a parameter known as the variance-power parameter (20). The Tweedie regression model is defined by the following components:

1. Response variable: The response variable is a continuous variable that follows a Tweedie distribution.
2. Linear predictor: This is a linear combination of predictor variables and their associated regression coefficients. It is defined as (21):
  η=Xβ (9)

The linear predictor (η) is a function that estimates the response variable using a set of predictor variables represented by a design matrix (X), and a vector of regression coefficients (β) that are multiplied by the predictor variables.

(1) Link function: This function is used to link the linear predictor to the response variable. In Tweedie regression, the canonical link function is used, which is defined as (22):
  $$g(\mu )=\{\begin{array}{cc} \mathrm{Log}(\mu ),\; & \;p=1\;\\ \frac{1}{1-p}[(\mu^{1-p})-1],\; & \;p\neq 1\; \end{array}$$ (10)
  where g(*μ*) is the link function, μ is the mean of the Tweedie distribution, and *p* is the Tweedie variance-power parameter.
(2) Variance function: This function is used to model the variance of the response variable as a function of its mean. In Tweedie regression, this function can be expressed as (22):
  $$\mathrm{Var}(y)=\{\begin{array}{cc} \phi \mu ,\; & \;p=1\;\\ \phi \mu^{p},\; & \;p\neq 1\; \end{array}$$ (11)
  where Var(y) denotes the response variance, μ is the mean, *φ* is the dispersion (scale) parameter, and *p* is the Tweedie variance-power parameter.
(3) Likelihood function: This function is used to estimate the model's parameters. In Tweedie regression, the likelihood function is defined as (23):
  $$L(\beta ,\phi ,p|y,X)=\prod_{i=1}^{n}\;f(y_{i}|x_{i};\beta ,\phi ,p)$$ (12)
  where *n* stands for the sample size, $f(y_{i}|x_{i};\beta ,\phi ,p)$ is the probability density function of the Tweedie distribution, and *y* and *X* are the vectors of response and predictor variables, respectively.

The parameters of the Tweedie regression model can be estimated using maximum likelihood estimation (MLE), in which the parameter values are determined by maximizing the likelihood of the observed data (16). Because a closed-form solution is generally unavailable, iterative numerical procedures, such as the Newton–Raphson algorithm or Fisher scoring, may be employed to estimate the regression coefficients and dispersion parameter (24).

### 3.3 Gaussian process regression (GPR)

Gaussian Process Regression (GPR) is a nonparametric Bayesian modeling technique that establishes a probabilistic relationship between a continuous target variable and a set of input features. A Gaussian process can be defined as a collection of random variables such that any finite subset of these variables follows a joint multivariate Gaussian distribution. Within the GPR framework, the unknown relationship between the inputs and the response is modeled as a latent function governed by a Gaussian process. This probabilistic formulation not only enables accurate estimation of the response but also provides a principled measure of predictive uncertainty for each estimated value (25).

For a regression problem, the observations can be expressed as

$$y_{i}=f(x_{i})+\varepsilon_{i},\;\varepsilon_{i}∼N(0,\sigma_{n}^{2}),$$ (13)

where f(x) is the unknown latent function and $\varepsilon_{i}$ represents independently distributed Gaussian noise with variance $\sigma_{n}^{2}$. A Gaussian-process prior is assigned to the latent function as

$$f(x)∼GP(m(x),k(x,x^{\prime })),$$ (14)

where m(x)=E[f(x)] denotes the mean function and $k(x,x^{\prime })=\mathrm{Cov}[\;f(x),f(x^{\prime })]$ is the covariance or kernel function. The kernel determines the similarity between different input observations and therefore controls important properties of the inferred function, including its smoothness and variability. Commonly adopted kernels include the squared-exponential, Matérn, rational-quadratic, and linear covariance functions (26–28).

The development of a GPR model involves the following principal stages (29, 30):

1. A suitable mean function and covariance kernel are selected according to the expected characteristics of the underlying input–output relationship.
2. The kernel parameters and observation-noise variance are estimated, commonly by maximizing the log marginal likelihood of the training data.
3. The posterior Gaussian distribution is computed for the test inputs by conditioning the prior process on the observed training data.
4. The posterior mean is used as the predicted response, whereas the posterior variance provides a quantitative measure of predictive uncertainty.

Consequently, GPR produces both point estimates and uncertainty intervals, making it particularly useful for regression problems involving limited experimental observations and complex nonlinear relationships (30).

### 3.4 K-nearest neighbors (KNN)

K-nearest neighbors (KNN) regression is a nonparametric, instance-based learning method that estimates the continuous response associated with a new input vector by exploiting the similarity between that observation and neighboring samples in the training dataset (31–33). Unlike parametric regression techniques, KNN does not assume a predefined functional relationship between the predictors and response. Instead, for a query point $x^{*}$, the algorithm identifies a neighborhood $N_{K}(x^{*})$ containing the *K* training observations closest to $x^{*}$ according to a selected distance metric. The unweighted KNN prediction is calculated as

$$\hat{y}(x^{*})=\frac{1}{K}\sum_{i\in N_{K}(x^{*})}y_{i},$$ (15)

where $y_{i}$ denotes the observed response of the *i* th neighboring sample. Alternatively, a distance-weighted prediction can be employed:

$$\hat{y}(x^{*})=\frac{\sum_{i\in N_{K}(x^{*})}w_{i}y_{i}}{\sum_{i\in N_{K}(x^{*})}w_{i}},\;w_{i}=\frac{1}{{\left[d(x^{*},x_{i})+\varepsilon \right]}^{p}},$$ (16)

where $d(x^{*},x_{i})$ is the distance between the query point and its *i*th neighbor, p>0 controls the influence of distance, and ε is a small positive constant that prevents division by zero (34). A schematic representation of the KNN prediction process is provided in Figure 2.

**Figure 2:**
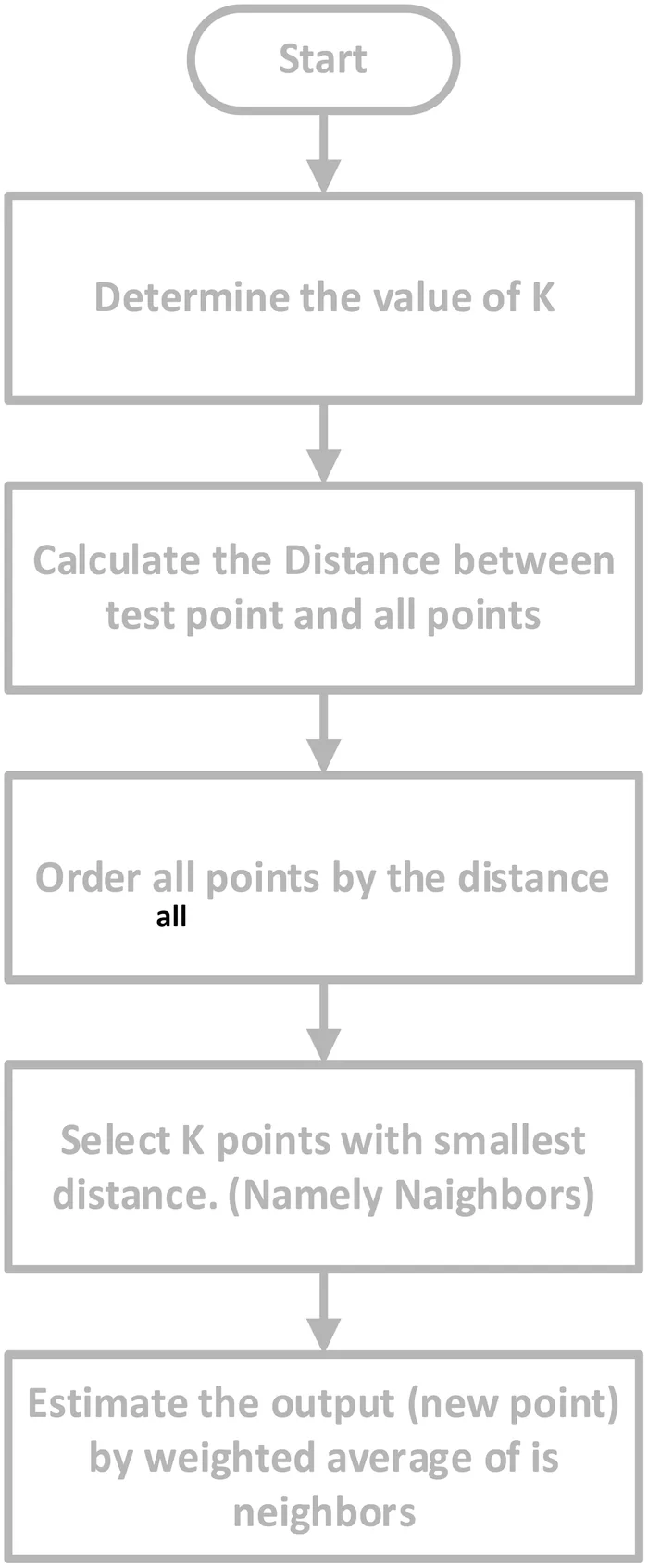
Prediction flowchart of KNN model.

### 3.5 Model-development and evaluation workflow

The model-development and evaluation sequence used in this study was as follows. First, the 40 experimental observations were organized using pressure and temperature as the two input variables and mole fraction and SC-CO_2_ density as separate target outputs. Second, GPR, TDR, and KNN regression models were fitted for each target. Third, candidate model settings were optimized with the Cheetah Optimizer by minimizing predictive error within the adopted model-development procedure. Fourth, the configured models generated predictions for the test data, and performance was quantified using *R*^2^, MSE, and MAE. Finally, the best-performing model was identified separately for each target, and the selected TDR and GPR models were used to visualize and interpret the effects of pressure and temperature. For the target-specific models used in the subsequent interpretation, the optimized TDR configuration was power = 2.5, alpha = 0.75, and maximum iterations = 2,600, whereas the optimized GPR configuration used alpha = 4.9233 × 10^−6^ with three optimizer restarts.

## 4 Results and discussion

The three regression models—GPR, KNN, and TDR—showed target-dependent predictive performance. For mole-fraction prediction, TDR achieved the highest *R*^2^ (0.98589) and the lowest MAE (4.34427 × 10^−7^), followed closely by GPR, whereas KNN showed lower accuracy. For density prediction, GPR clearly provided the strongest performance, with *R*^2^ = 0.99155 and MAE = 1.70910 × 10^1^; KNN ranked second and TDR showed the largest errors. The corresponding results are summarized in Tables 2, 3. Because mole fraction and density are expressed on very different numerical scales, the absolute MSE and MAE values should be interpreted within each target rather than compared directly across the two outputs. The different model rankings also indicate that the response structure is target-specific: the positive, strongly varying mole-fraction response is represented effectively by the Tweedie formulation, while the smoother nonlinear density surface is particularly well suited to GPR.

**Table 2:** Results for mole-fraction prediction.

Algorithm	*R* ^2^	MSE	MAE
GPR	0.98369	5.1166 × 10^−13^	5.05411 × 10^−7^
KNN	0.87344	2.1221 × 10^−12^	8.33079 × 10^−7^
TDR	0.98589	3.1529 × 10^−13^	4.34427 × 10^−7^

**Table 3:** Results for solvent density prediction.

Algorithm	*R* ^2^	MSE	MAE
GPR	0.99155	4.7169 × 10^2^	1.70910 × 10^1^
KNN	0.94785	2.8253 × 10^3^	4.16926 × 10^1^
TDR	0.80368	7.0016 × 10^3^	6.81962 × 10^1^

Figures 3, 4 compare the experimental and predicted values for mole fraction and density, respectively, for all three models. The numerical and graphical results consistently identify TDR as the preferred model for mole-fraction prediction and GPR as the preferred model for density prediction. The strong TDR result for mole fraction (*R*^2^ = 0.98589; MAE = 4.34427 × 10^−7^) is consistent with the flexibility of the Tweedie family for positive continuous responses whose variance can change with the mean. In contrast, GPR is advantageous for the density target because its kernel-based probabilistic formulation can represent a smooth nonlinear response surface from a relatively small experimental dataset. Therefore, the subsequent physical-response analysis uses TDR for mole fraction and GPR for density (16).

**Figure 3:**
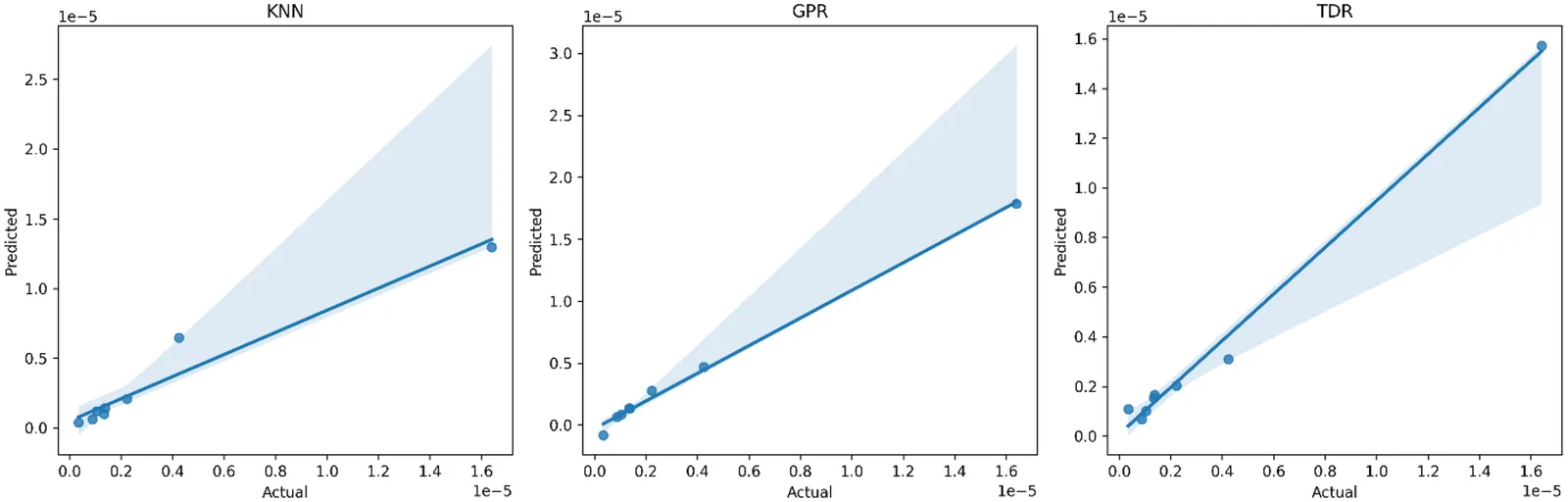
Fitting charts for mole fraction test data (all models).

**Figure 4:**
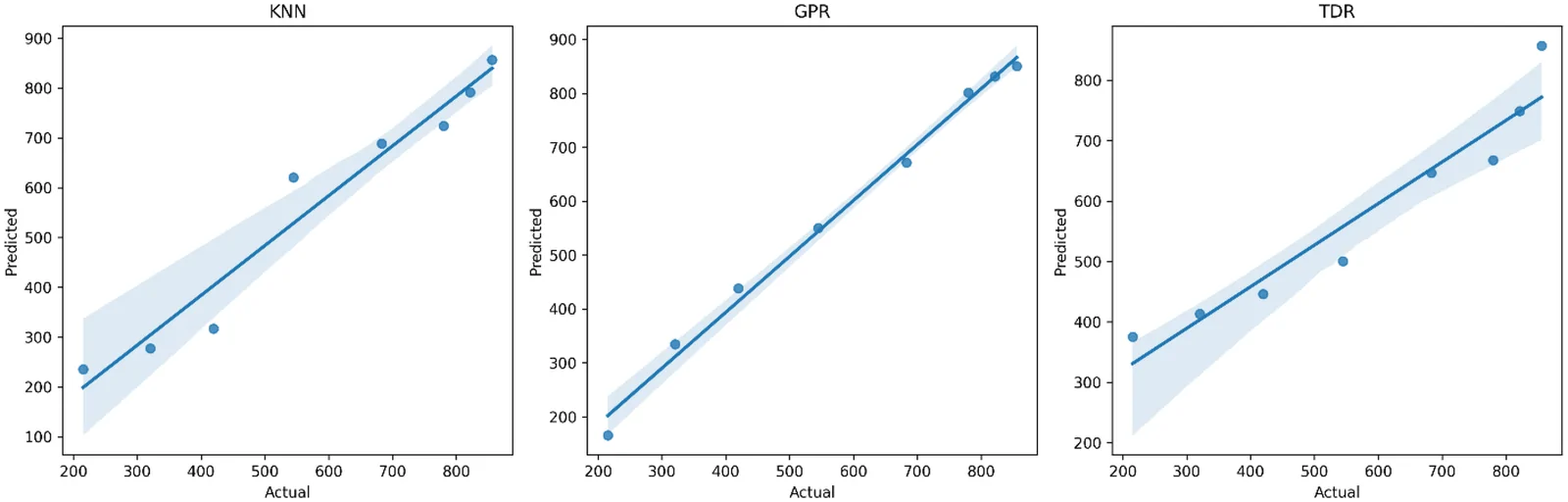
Fitting charts for density test data (all models).

### 4.1 TDR model for mole fraction

The final TDR model for paracetamol mole-fraction solubility used power = 2.5, alpha = 0.75, and a maximum of 2,600 iterations, as summarized in the methodological workflow. To visualize the individual effects of temperature and pressure, two-dimensional response curves were generated by varying one input while holding the other at several fixed levels. Figures 5, 6 present these single-factor views, and Figure 7 presents their combined response surface.

**Figure 5:**
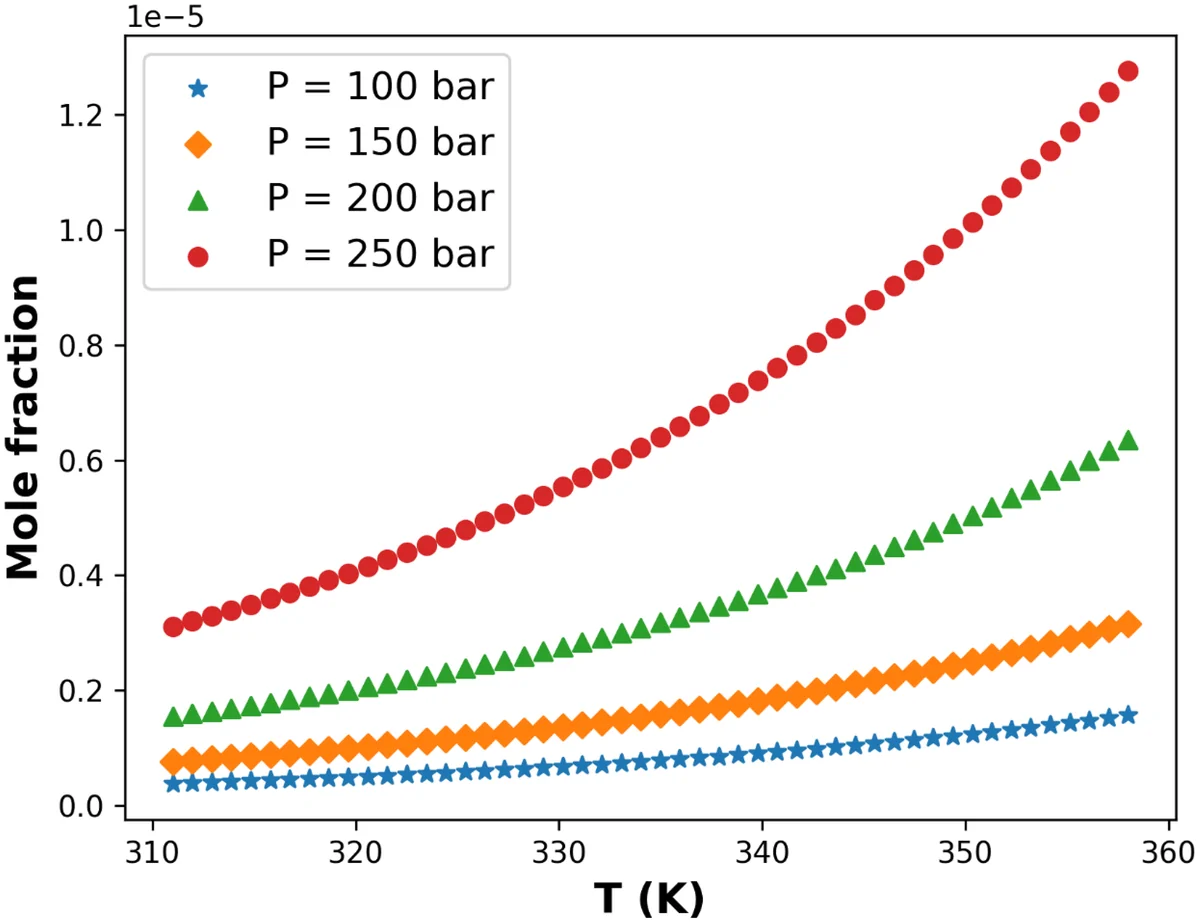
Individual effect of T (K) on mole fraction.

**Figure 6:**
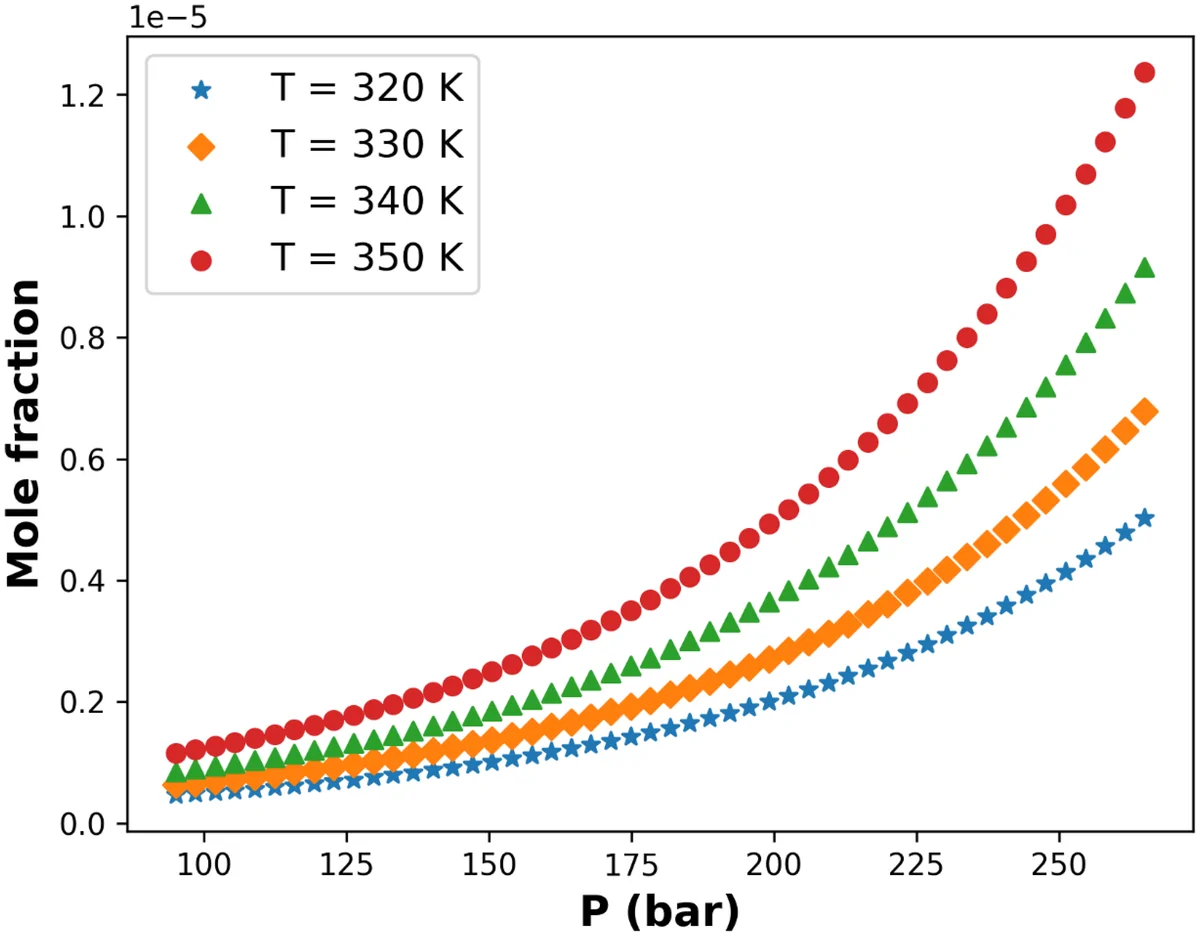
Individual effect of P (bar) on mole fraction.

**Figure 7:**
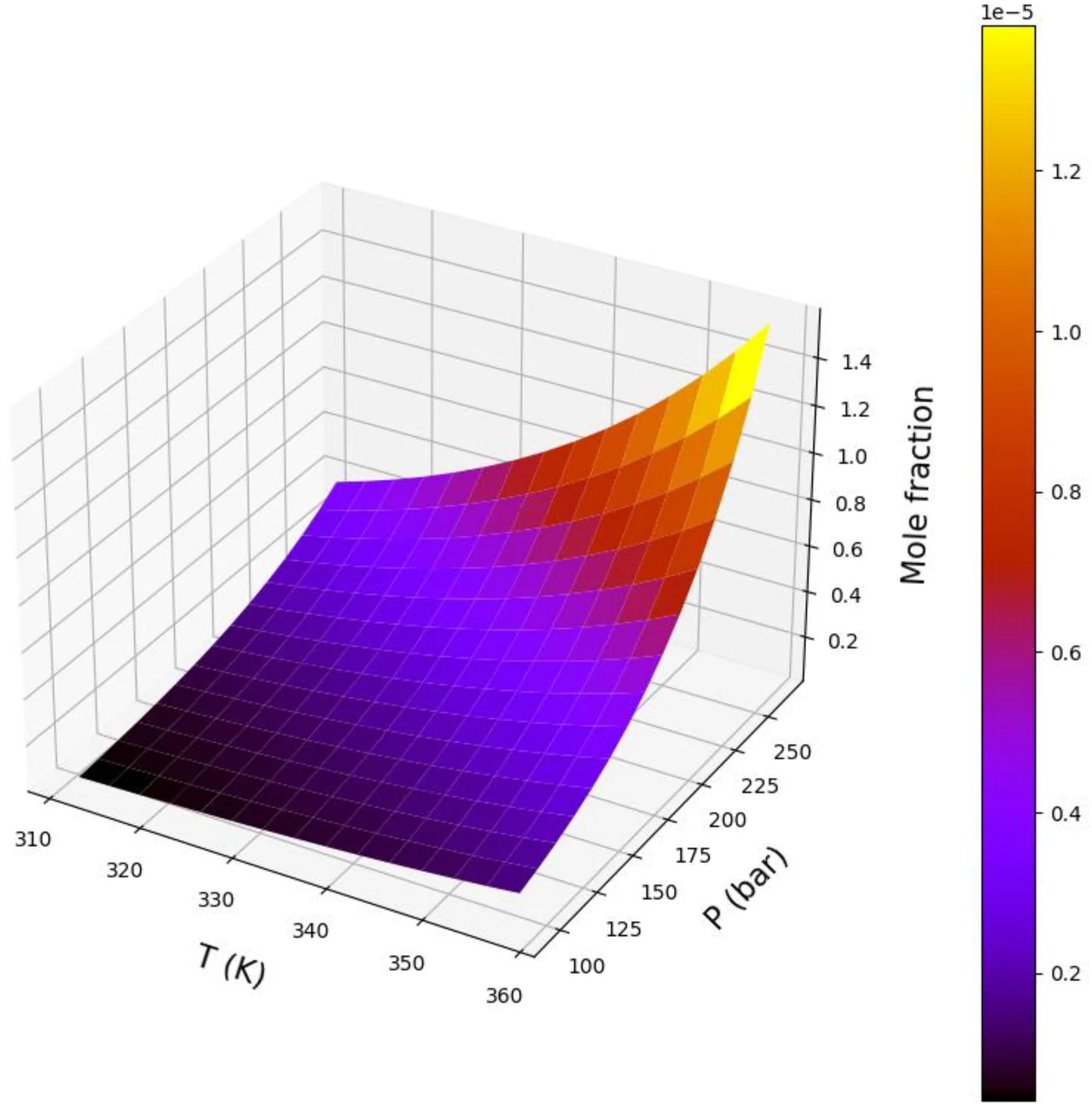
Combined effect of inputs on mole fraction.

Figure 5 shows the TDR-predicted temperature dependence of paracetamol mole-fraction solubility at pressures from 100 to 250 bar, while Figures 6, 7 show the corresponding pressure dependence and combined pressure–temperature response. Within the investigated domain, the predicted mole fraction increases with temperature at each plotted pressure and increases with pressure at each plotted temperature. This model behavior is physically interpretable through the competing effects of temperature: increasing temperature tends to decrease SC-CO_2_ density and solvent power, while simultaneously increasing solute vapor pressure. For the pressure and temperature ranges examined here, the positive temperature response in Figures 5–7 indicates that the vapor-pressure contribution dominates the density-reduction effect. This interpretation is limited to the investigated operating domain and should not be extrapolated as evidence of the same temperature dependence outside that range. The increasing separation of the pressure curves at higher temperatures further indicates a coupled pressure–temperature influence. Alotaibi et al. (16) revealed similar results for paracetamol solubility as well as density of solvent.

Figure 7 integrates the two input effects in a three-dimensional TDR response surface. The rise in mole fraction with pressure is consistent with compression of SC-CO_2_, which increases fluid density and generally strengthens its solvent power. The simultaneous temperature effect reflects the balance described above rather than a single density-controlled mechanism (16). Accordingly, the model-predicted solubility surface should be interpreted together with the density trends discussed in Section 4.2.

### 4.2 GPR model for density analysis

The final GPR model for density used alpha = 4.9233 × 10^−6^ and three optimizer restarts. Figures 8, 9 show the individual effects of temperature and pressure, and Figure 10 presents the combined three-dimensional density surface. The results match the outputs reported by Alotaibi et al. (16) for prediction of paracetamol. The GPR predictions reproduce the expected qualitative pattern within the studied domain: SC-CO_2_ density decreases with increasing temperature and increases with increasing pressure. This agreement supports the physical consistency of the model predictions within the investigated range; it does not, by itself, establish general model validity or guarantee reliable extrapolation beyond the available data (16).

**Figure 8:**
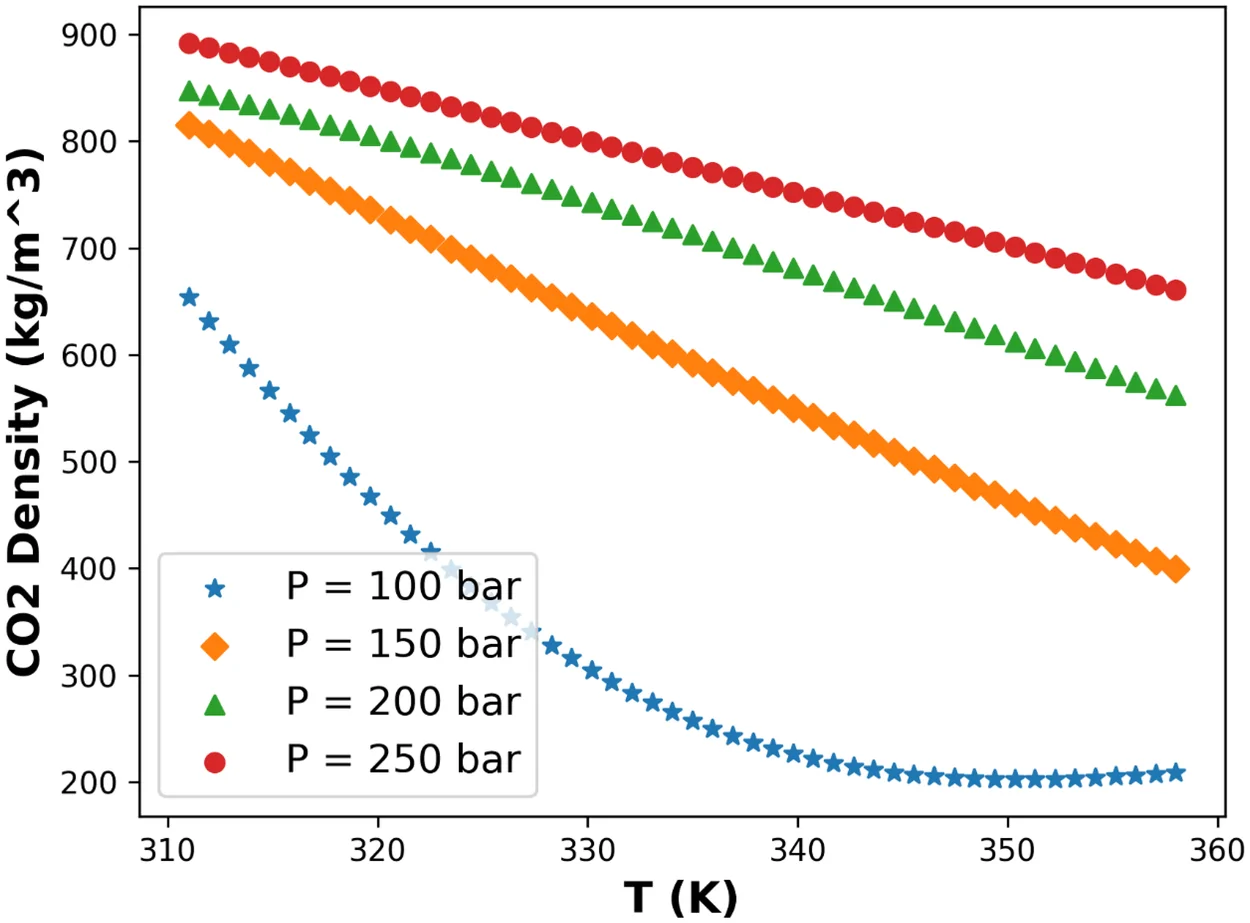
Individual effect of T (K) on density.

**Figure 9:**
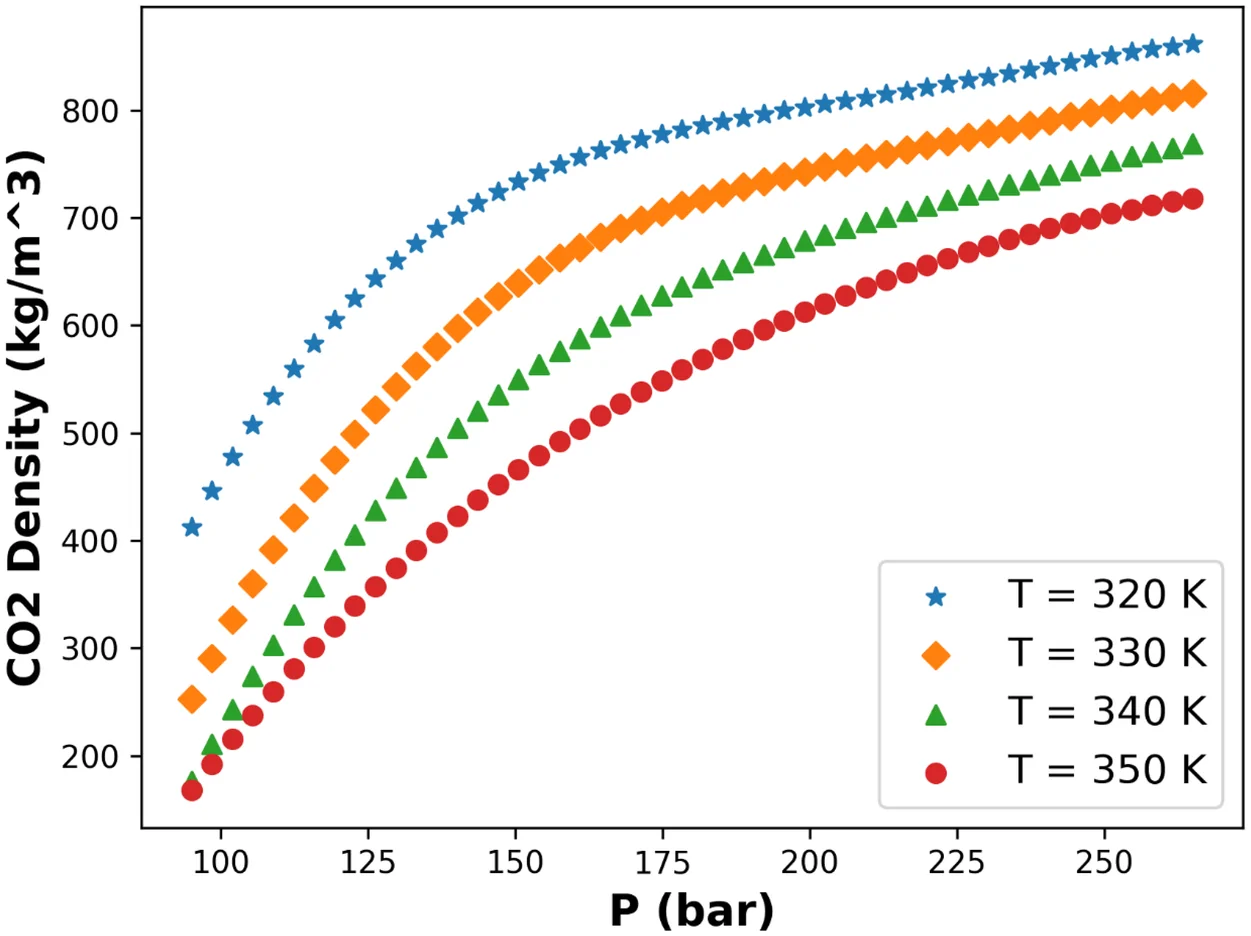
Individual effect of P (bar) on density.

**Figure 10:**
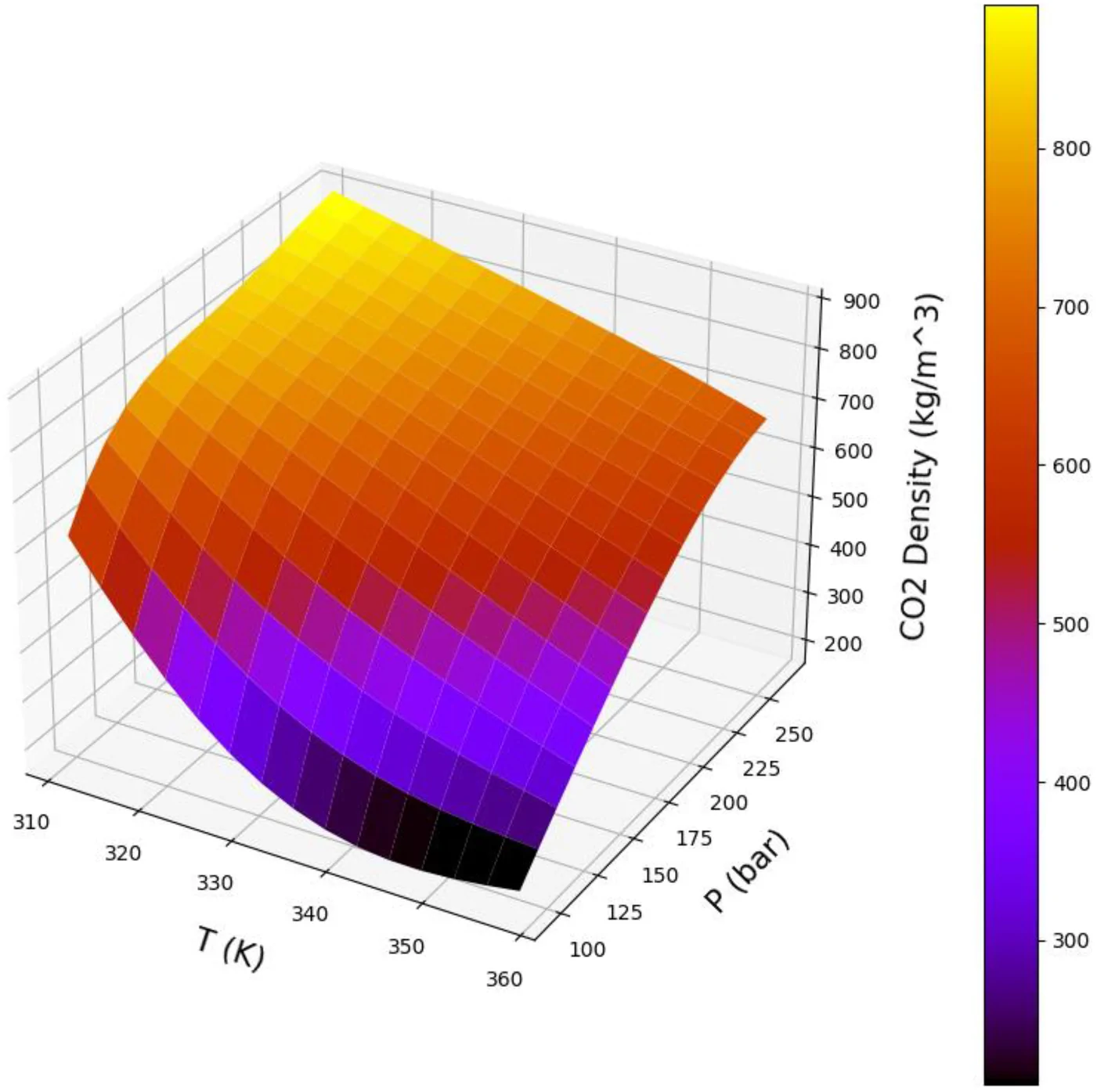
Combined effect of inputs on density.

Figure 9 shows that the GPR-predicted SC-CO_2_ density increases monotonically and nonlinearly with pressure over the plotted temperature range of 320–350 K. The increase is steeper at lower pressures and gradually flattens as pressure rises, which is consistent with the decreasing compressibility of the fluid as it becomes denser. Temperature produces the opposite trend: at a fixed pressure, density decreases as temperature increases because of thermal expansion. For example, near 100 bar the plotted density decreases from roughly 450 kg m^−3^ at 320 K to about 190 kg m^−3^ at 350 K, whereas near 250 bar it decreases from approximately 850 to 700 kg m^−3^. These density trends directly complement the solvent-power interpretation in Section 4.1: increasing pressure generally strengthens the solvating capacity of SC-CO_2_ by increasing density, whereas increasing temperature can weaken the density contribution to solvent power. The positive temperature dependence of paracetamol solubility predicted in Figures 5–7 therefore indicates that, within the present domain, the opposing increase in solute vapor pressure is sufficiently strong to outweigh the density decrease. GPR also achieved the best density metrics among the tested models (*R*^2^ = 0.99155 and MAE = 1.70910 × 10^1^), consistent with its ability to represent a smooth nonlinear function using a kernel-based model that is suitable for small datasets (16).

## 5 Conclusion

This study developed an AI-assisted predictive framework that integrates machine-learning regression with Cheetah Optimizer-based hyperparameter tuning for paracetamol mole-fraction solubility and SC-CO_2_ density. Using 40 experimental observations with pressure and temperature as the two inputs, GPR, KNN, and TDR were evaluated using *R*^2^, MSE, and MAE. TDR provided the best mole-fraction prediction (*R*^2^ = 0.98589; MAE = 4.34427 × 10^−7^), whereas GPR provided the best density prediction (*R*^2^ = 0.99155; MAE = 1.70910 × 10^1^). The target-dependent ranking demonstrates that model selection should be performed separately for each predicted property rather than assuming that one algorithm is universally optimal. The pressure- and temperature-response analyses were also physically consistent with the expected competition between SC-CO_2_ density and solute vapor-pressure effects within the studied range. The principal limitations are the modest dataset size (40 observations), the use of only two input variables, and the restriction of the analysis to the investigated pressure–temperature domain. Accordingly, the models are most appropriately used for interpolation and preliminary SC-CO_2_ process assessment within conditions represented by the data; additional experimental observations and external validation would be required before broader extrapolation or process-wide generalization.

## Data Availability

The original contributions presented in the study are included in the article/Supplementary Material, further inquiries can be directed to the corresponding author/s.
